# Supplementary material for: Damage-induced reactive oxygen species regulate vimentin and dynamic collagen-based projections to mediate wound repair
Source: eLife. 2018 Jan 16;7:e30703. doi: 10.7554/eLife.30703 (PMC5790375; doi:10.7554/eLife.30703)
Supplement: Figure 2—source data 1. [file elife-30703-fig2-data1.docx]

Sequence of Commands used in R for LSMeans analysis:

install.packages("lsmeans")

library(lsmeans)

data=read.table("location of your data/data1.txt",header=T)

replicate=as.factor(data$replicate)

condition=as.factor(data$condition)

measure=data$measure

fit=lm(measure~replicate+condition)

LSMeans=lsmeans(fit,"condition") #Calculate the adjustment means for the different conditions

LSMeans

Comparisons=contrast(LSMeans, "revpairwise") #Conducts pairwise comparisons between conditions

Comparisons
